# Amygdala-centred functional connectivity affects daily cortisol concentrations: a putative link with anxiety

**DOI:** 10.1038/s41598-017-08918-7

**Published:** 2017-08-16

**Authors:** Yuko Hakamata, Shotaro Komi, Yoshiya Moriguchi, Shuhei Izawa, Yuki Motomura, Eisuke Sato, Shinya Mizukami, Yoshiharu Kim, Takashi Hanakawa, Yusuke Inoue, Hirokuni Tagaya

**Affiliations:** 1 Department of Adult Mental Health, National Institute of Mental Health, National Center of Neurology and Psychiatry, Tokyo, Japan; 2 Department of Health Sciences, Kitasato University School of Allied Health Sciences, Kanagawa, Japan; 3 Department of Radiology, Kitasato University Hospital, Kanagawa, Japan; 4 Integrative Brain Imaging Center, National Center of Neurology and Psychiatry, Tokyo, Japan; 5 Occupational Stress Research Group, National Institute of Occupational Safety and Health, Kanagawa, Japan; 6 Department of Psychophysiology, National Institute of Mental Health, National Center of Neurology and Psychiatry, Tokyo, Japan; 7 Department of Medical Radiological Technology, Kyorin University School of Health Sciences, Tokyo, Japan; 8 Department of Clinical Engineering, Kitasato University School of Allied Health Sciences, Kanagawa, Japan; 9 Department of Diagnostic Radiology, Kitasato University School of Medicine, Kanagawa, Japan

## Abstract

The amygdala plays a critical role in emotion. Its functional coupling with the hippocampus and ventromedial prefrontal cortex extending to a portion of the anterior cingulate cortex (ACC) is implicated in anxiogenesis and hypothalamic-pituitary-adrenal (HPA) system regulation. However, it remains unclear how amygdala-centred functional connectivity (FC) affects anxiety and cortisol concentrations in everyday life. Here, we investigate the relationship between daily cortisol concentrations (dCOR) and amygdala-centred FC during emotional processing in forty-one healthy humans. FC analyses revealed that higher dCOR predicted strengthened amygdala-centred FC with the hippocampus and cerebellum, but inhibited FC with the supramarginal gyrus and a perigenual part of the ACC (pgACC) when processing fearful faces (vs. neutral faces). Notably, the strength of amygdala-hippocampus FC mediated the positive relationship between cortisol and anxiety, specifically when the effect of amygdala-pgACC FC, a presumptive neural indicator of emotional control, was taken into account. Individuals with diminished connectivity between the amygdala and pgACC during fear-related processing might be more vulnerable to anxiogenesis as it pertains to greater circulating cortisol levels in everyday life. Individual functional patterns of amygdala-hippocampal-pgACC connectivity might provide a key to understand the complicate link between cortisol and anxiety-related behaviors.

## Introduction

The hypothalamic-pituitary-adrenal (HPA) axis is an endocrine system that helps the organism adjust to internal and external environmental challenges. Glucocorticoid (cortisol in humans), its end hormonal product, plays a critical role in regulating the HPA axis through its major receptors in the central nervous system (CNS): glucocorticoid receptors (GRs) and mineralocorticoid receptors (MRs). GRs are expressed throughout the brain, while MRs are more restricted. However, both exist in the hippocampus (HC) and amygdala^1, 2^, indicating the importance of these 2 limbic structures in HPA axis regulation.

The amygdala is known to stimulate corticotropin releasing hormone (CRH)-producing neurons in the hypothalamic paraventricular nucleus (PVN) via intermediating neurons in the bed nucleus of the stria terminalis and other hypothalamic adjacent nuclei, interacting with the HC and medial prefrontal cortex (mPFC)^3^. Interestingly, this pathway becomes activated when an organism anticipates a potential threat arriving, but not necessarily when a salient threat actually arrives^3^. Indeed, previous meta-analytic functional magnetic resonance imaging (fMRI) studies have confirmed that the amygdala responds to fearful faces (that are not themselves imminent dangers) with markedly increased activity^4–6^. Moreover, its exaggerated responses and disrupted functional connectivity (FC), which is characterized by diminished emotional regulatory control of the mPFC [specifically its ventral part (vmPFC) comprised of the medial orbitofrontal cortex (mOFC) extending to a portion of the anterior cingulate cortex (ACC)], have been corroborated as prominent features in patients with emotional dysregulations such as depression and anxiety^7, 8^. Such increased amygdala responses to emotional stimuli have been reported in healthy participants as well, especially when their cortisol levels were elevated by stress^9, 10^ or pharmacological manipulation^11, 12^, though several contrary findings have been reported^13, 14^. Additionally, the magnitudes of stress- and drug-induced cortisol responses are known to be inversely correlated with amygdala-HC FC strength during rest^15, 16^ and positively correlated with amygdala-mPFC FC while processing emotional faces^13^. Despite the conceivable link between cortisol and emotional dysregulation mediated via the CNS, it remains unclear how amygdala-centred FC is involved in the relationship between cortisol and anxiety/depression.

In contrast to externally stimulated cortisol, the literature investigating crosstalk between endogenous cortisol and the amygdala and its neural connectivity is surprisingly limited, notwithstanding the evidence indicating that unstimulated cortisol is a key neurobiological component of emotional dysregulations associated with anxiety and depression^17^. Some fMRI studies have reported that greater baseline cortisol levels, measured from 10 to tens of minutes before MRI with experimental tasks, predicted attenuated amygdala responses to fearful faces (vs. happy faces)^10^ and oppositely heightened amygdala responses to emotional faces (vs. animals)^18^. Similarly, higher baseline cortisol was associated with more diminished FC between the amygdala and perigenual ACC (pgACC) during rest^19^, although participants’ tension and apprehension might have boosted baseline cortisol levels. In contrast, an fMRI study using saliva cortisol collected at home on non-scanning days has found that greater (normative) diurnal cortisol decline showed diminished amygdala activity and enhanced vmPFC activity when participants attempted to allay their negative affect^20^. However, no study has so far probed how task-irrelevant unstimulated cortisol affects amygdala-centred FC during emotional processing, and more specifically how FC between the amygdala, HC, and vmPFC is involved in a link between unstimulated cortisol and anxiety/depression.

We therefore investigated the effect of daily circulating cortisol levels on amygdala-centred FC during an emotional faces perception task using saliva gathered in task-irrelevant contexts for 2 consecutive typical weekdays, which provides reliable estimates of baseline cortisol levels^21^. We hypothesized that, while processing fearful faces (vs. neutral faces), FC between the amygdala, HC, and vmPFC would be significantly associated with task-irrelevant cortisol as well as anxiety/depression. Provided that amygdala-HC-vmPFC FC was significantly correlated with both cortisol and anxiety/depression, we further examined a possible mediation effect of the amygdala-HC-vmPFC FC on the relationship between cortisol and anxiety/depression.

## Results

### Cortisol data

Salivary cortisol data is shown in Table 1. With the exception of DS, all cortisol measures failed the Shapiro-Wilk test and, consequently, were square root transformed. No cortisol measures were significantly associated with age, sex, menstrual period, BMI, monthly alcohol consumption, years of education, sleep duration, sleep quality, perceived stress, or time of awakening during the 2 saliva collection days (see Supplementary material and Table S1). Due to the great commonality between dCOR and AUCg (Table S1; *r* = 0.96, *p* < 0.001) and their overlapping results, we focused on dCOR in the following analyses.

**Table 1:** Salivary cortisol data (nmol/L).

	Mean	SD
Time 1 (at awakening)	14.90	9.55
Time 2 (30 min after awakening)	24.45	10.81
Time 3 (bedtime)	4.15	4.96
dCOR	43.51	22.47
AUCg	259.94	137.77
CAR	2.39	2.00
DS	10.75	6.62

Abbreviations: SD, standard deviation; dCOR, daily cortisol concentrations; AUCg, area under the curve with respect to ground (at 3 points); CAR, cortisol awakening response (i.e., area under the curve with respect to increase); DS, diurnal slope.

### Amygdala-centred FC during fearful faces processing

**Figure 1:**
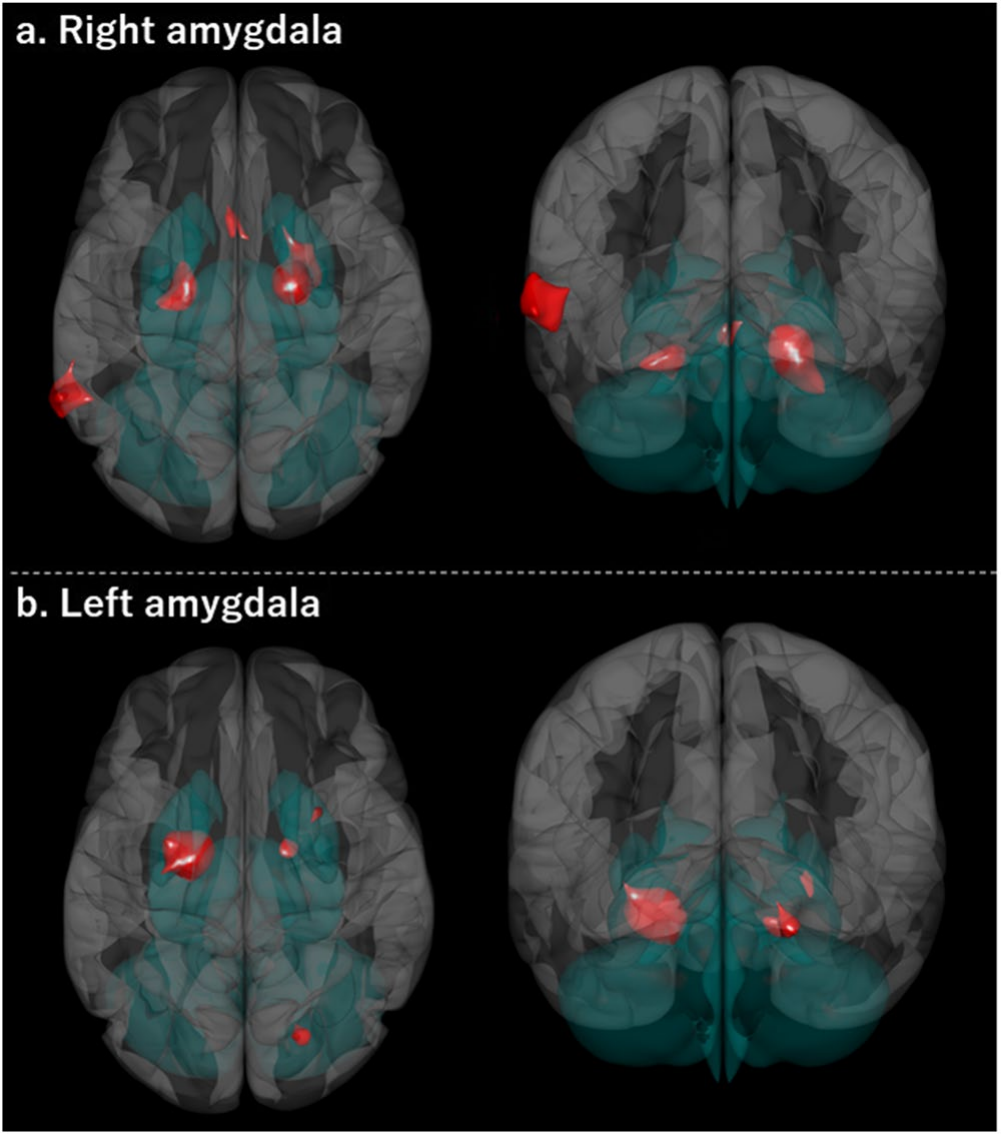
Amygdala-centred functional connectivity during fearful faces processing. Seed region: the amygdala that showed significantly increased activation in response to fearful faces (vs. neutral faces) in this sample (L-Amy: MNI −21 −10 −17, 351 mm^3^, R-Amy: MNI 21 −10 −17, 189 mm^3^), which are comprised of the caudomedial portion of the amygdala. Clusters were significant at FDR-corrected *p* < 0.05 [both height threshold and cluster threshold (cluster-size correction)]. Abbreviations: L-Amy, left amygdala; R-Amy, right amygdala; MNI, Montreal Neurological Institute space; FDR, false-discovery rate.

FC results for the simple effect of fearful faces in the entire sample are presented in Fig. 1 for each side of the amygdala seed. Both R-Amy and L-Amy showed increased HC connectivity (Table 2).

**Table 2:** Amygdala-centred functional connectivity during fearful faces processing

Region	Side	voxels	MNI coordinate			*T*
			x	y	z	
*Right*						
Parahippocampal region (including a part of the amygdala and the hippocampus)	R	441	20	−8	−16	17.77
Hippocampus	L	119	−20	−8	−18	6.98
Middle temporal gyrus	L	47	−70	−52	−2	5.85
Subcallosal gyrus	L	29	−2	12	−10	6.41
*Left*						
Parahippocampal region (including a part of the amygdala, hippocampus, putamen, and pallidum)	L	648	−16	−6	−14	22.30
Occipital fusiform gyrus (including a part of the cerebellum)	R	100	18	−4	−18	6.58
Parahippocampal region (including a part of the amygdala and hippocampus)	R	88	20	−76	−18	6.27
Putamen	R	60	28	8	−6	6.26

Seed region is the amygdala whose cluster showed significantly increased activation in response to fearful faces (vs. neutral faces): L-Amy: MNI −21–10–17, 351 mm^3^, R-Amy: MNI 21 -10 -17, 189 mm^3^. These clusters are comprised of the caudomedial portion of the amygdala. Significant at whole-brain FDR-corrected *p* < 0.05.

#### Whole-brain analysis

Whole-brain analyses revealed that higher dCOR levels predicted significantly increased R-Amy-centred FC with the left HC (*T* = 4.75, FDR-corrected *p* = 0.004) and the left cerebellum (*T* = 3.55, FDR-corrected *p* = 0.047), and decreased FC with an anterior part of the left supramarginal gyrus (SMG) (*T* = −3.72, FDR-corrected *p* = 0.043) upon the appearance of fearful faces (vs. neutral faces). No other FC analyses showed significant relationships with cortisol indices other than CAR. The results of CAR are shown in Supplementary Material.

#### Seed-to-voxel analysis

**Figure 2:**
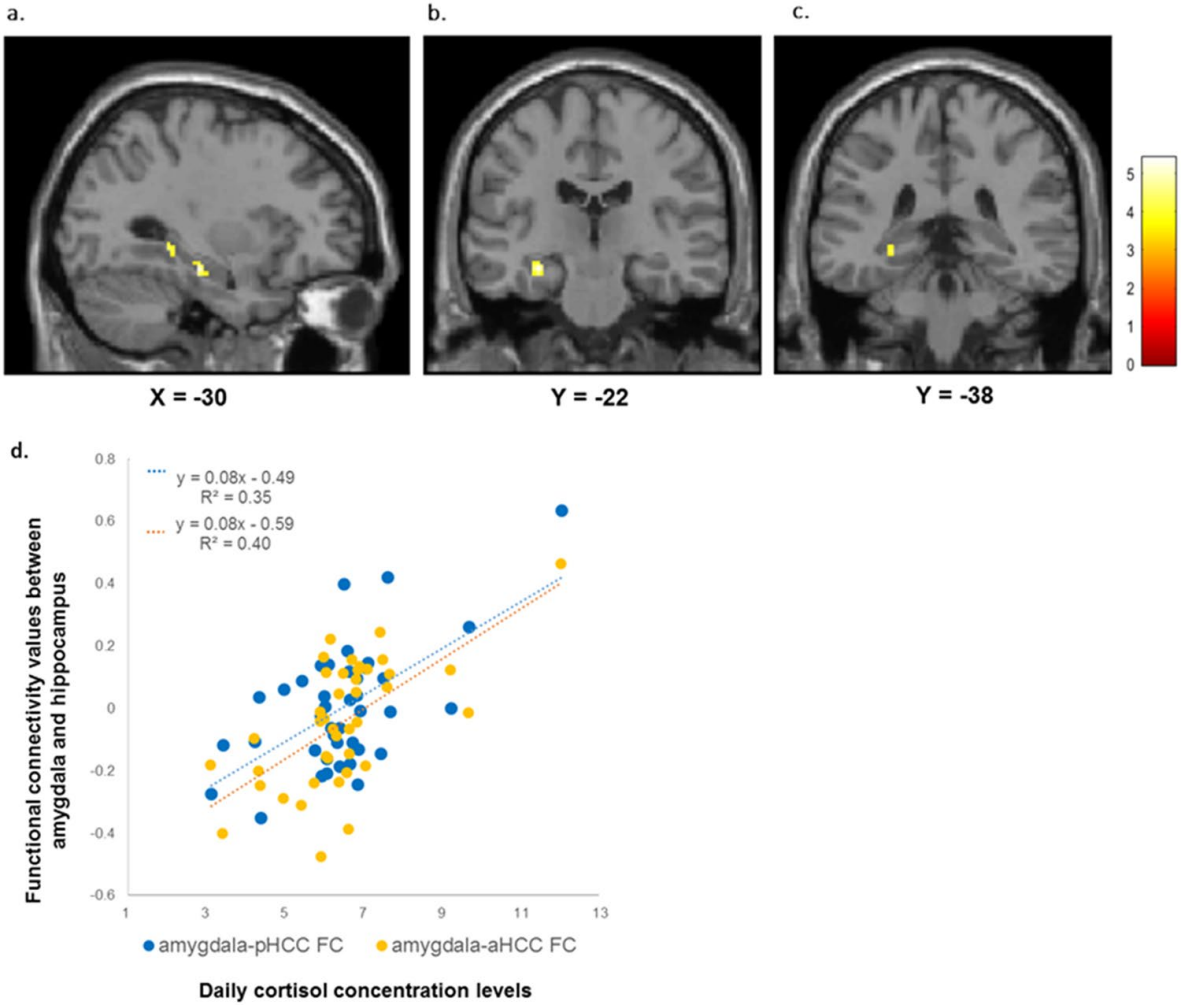
The HC displays greater FC with the amygdala during the perception of fearful faces (vs. neutral faces). (**a**) Significantly heightened FC between the amygdala and 2 different clusters within the HC (its strength parallels dCOR levels); (**b**) Anterior part of the HC (MNI −30 −22 −16, 280 mm^3^, *T* = 5.41, *p* < 0.001); (**c**) Posterior part of the HC (MNI −30 −38 −4, 144 mm^3^, *T* = 4.30, *p* < 0.001). These clusters were also significant at FWE-corrected *p* < 0.05 with SVC. Colour bar indicates T-value. (**d**) Scatter plot of the relationship between dCOR and amygdala-HC FC values in fearful faces processing (vs. neutral faces). Amygdala-pHCC FC values (blue circle). Amygdala-aHCC FC values (orange circle). Regression line of amygdala-pHCC FC (blue dotted line). Regression line of amygdala-aHCC FC (orange dotted line). Abbreviations: HC, hippocampus; FC, functional connectivity; dCOR, daily cortisol concentrations; MNI, Montreal Neurological Institute space; SVC, small volume correction; pHCC, a posterior part of the HC; aHCC, an anterior part of the HC.

Seed-to-voxel analysis further specified that 2 different clusters within the HC were more strongly connected with R-Amy as a function of dCOR: 1 was located on its anterior part [anterior HC (aHC): MNI −30 −22 −16, 280 mm^3^, *T* = 5.41, *p* < 0.001] (Fig. 2a,b) and the other was on its posterior part [posterior HC (pHC): MNI −30 −38 −4, 144 mm^3^, *T* = 4.30, *p* < 0.001] (Fig. 2a,c). A scatter plot of these clusters is presented in Fig. 2d.

**Figure 3:**
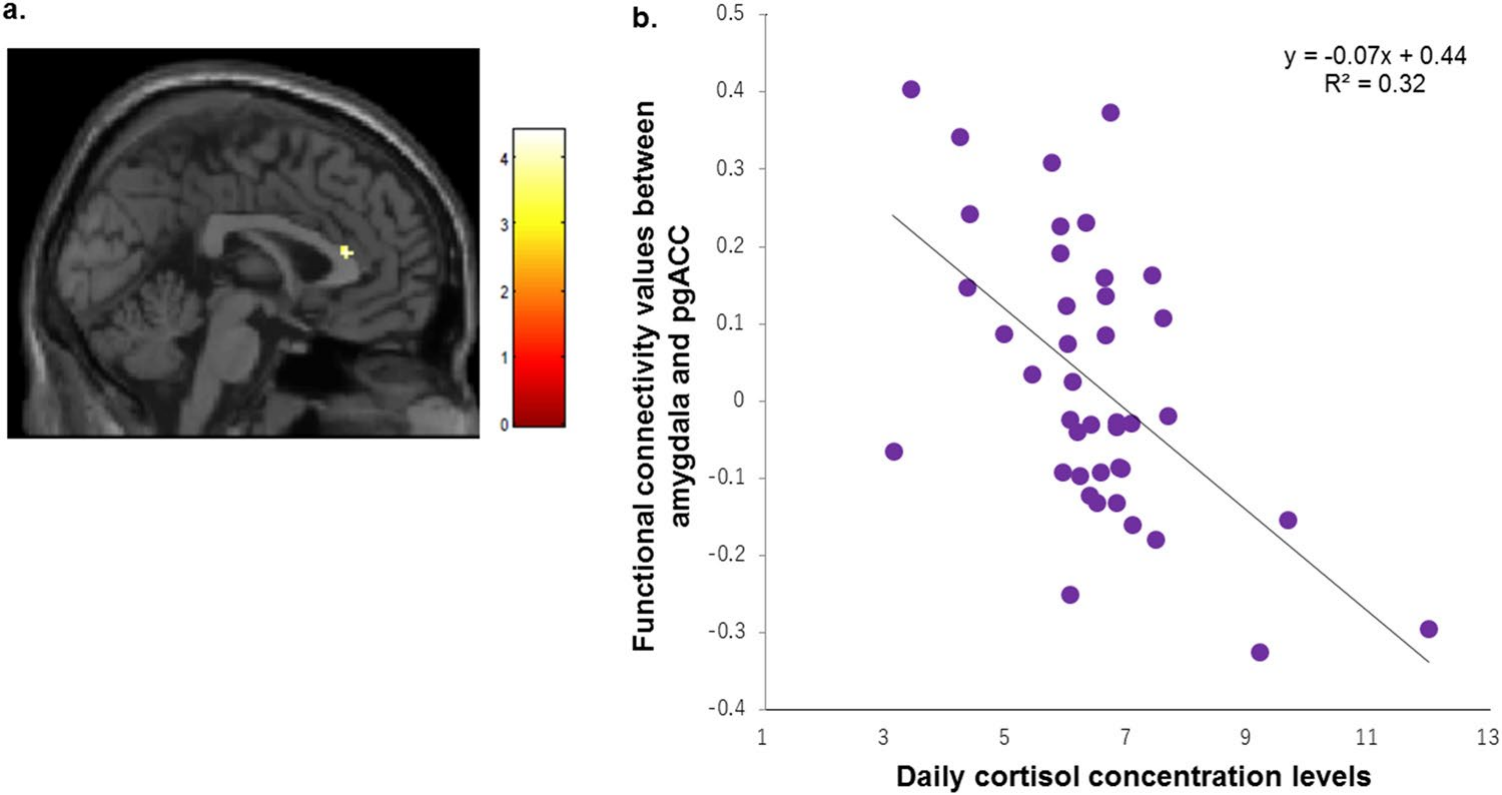
The pgACC exhibits diminished FC with the amygdala. (**a**) pgACC cluster found at MNI 0 30 14 (120 mm^3^; *T*  = 4.40, *p* < 0.001). Colour bar indicates T-value. (**b**) Scatter plot of the relationship between dCOR and amygdala-pgACC FC values in fearful faces processing (vs. neutral faces). Abbreviations: pgACC, perigenual anterior cingulate cortex; FC, functional connectivity; MNI, Montreal Neurological Institute space; dCOR, daily cortisol concentrations.

Furthermore, we found significant FC between the R-Amy and pgACC in the seed-to-voxel analysis (MNI 0 30 14, 120 mm^3^, *T* = 4.40, *p* < 0.001) (Fig. 3a), although it was not found in the whole-brain analysis possibly because the ROI of vmPFC was too extensive and functionally heterogeneous to detect a significance of β values averaged across all voxels therein. The scatter plot of the pgACC is presented in Fig. 3b. Thus, the amygdala-pgACC FC was factored into subsequent analyses.

### Regression analysis: amygdala-centred FC and anxiety/depression

Step-wise regression analyses demonstrated that R-Amy-pHC FC was the sole significant predictor for somatization symptoms [*R* ^2^ = 0.10, *F*(1,39) = 4.16, *p* = 0.049, *β* = 0.31] and anxiety symptoms [*R* ^2^ = 0.15, *F*(1,39) = 6.75, *p* = 0.013, *β* = 0.38], respectively. No other amygdala-centred FC was significantly associated with any HSCL symptom.

### Mediation analysis: effect of amygdala-centred FC on the relationship between cortisol and anxiety/depression

**Figure 4:**
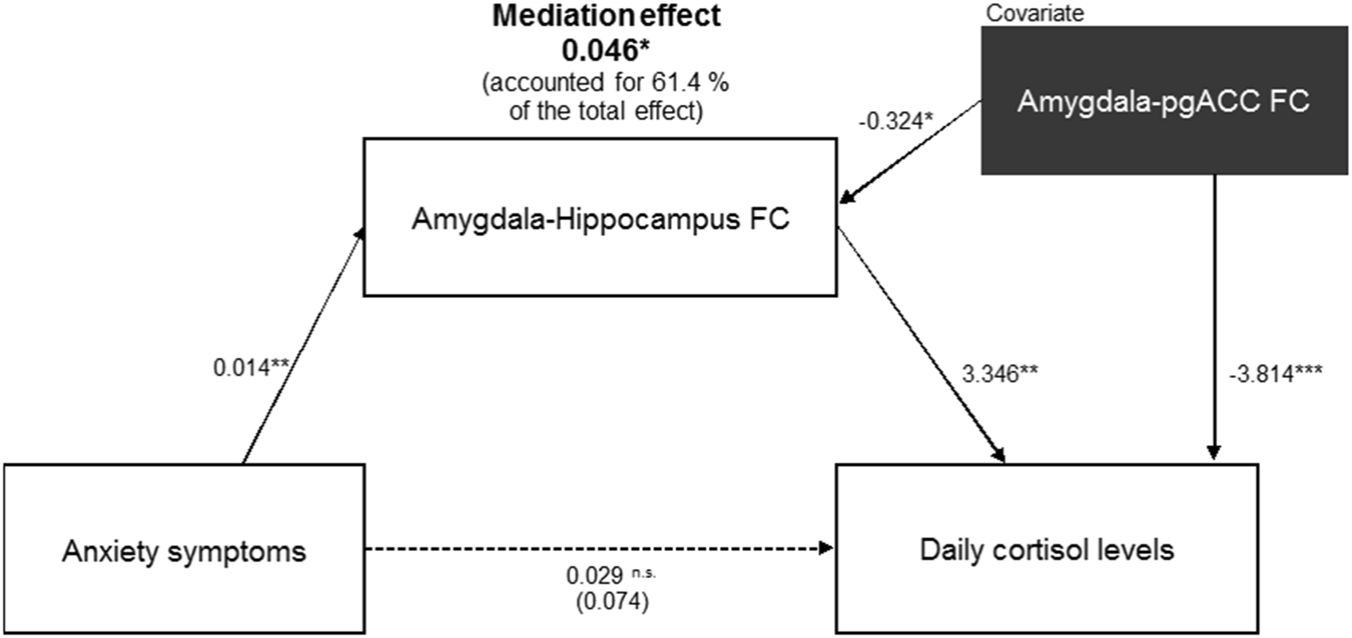
Results of mediation analysis. Amygdala-HC FC mediating effect on the relationship between anxiety symptoms and dCOR. The effect of the amygdala-pgACC FC was incorporated as covariate in the mediation model. Abbreviations: HC, hippocampus; FC, functional connectivity; dCOR, daily cortisol concentrations; pgACC, periginual anterior cingulate cortex. *p < 0.05, **p < 0.01, ***p < 0.001. All path coefficients are unstandardized regression weights. The value in parentheses indicates anxiety symptoms’ total effect on dCOR.

Due to amygdala-pHC FC’s significant correlation with both cortisol and anxiety, we examined whether it would mediate the relationship between anxiety symptoms and dCOR. At first, the simple correlation coefficients between anxiety (X) and dCOR (Y) were not significant (*r* = 0.26, *p* = 0.102). However, a significant direct effect of anxiety symptoms on dCOR emerged when the effect of amygdala-pgACC FC was factored in as a covariate [unstandardized regression coefficient (*B*) = 0.07, *p* = 0.040]. Furthermore, when amygdala-pHC FC was assigned as M, we observed a significant indirect effect of amygdala-pHC FC on the relationship between dCOR and anxiety symptoms [*B* = 0.05, 95% Cl = 0.00–0.12; standardized regression coefficient (*β*) = 0.20, 95% Cl =f 0.00–0.50; and *p* = 0.040] (Fig. 4). The statistical significance of the direct effect disappeared in the mediation model (*B* = 0.03, *p* = 0.40), indicating complete mediation by the amygdala-pHC FC. The mediation effect accounted for 61.4% of the anxiety symptoms’ total effect on dCOR.

## Discussion

We hypothesized that FC between the amygdala, HC, and vmPFC in emotional processing would be significantly associated with task-irrelevant cortisol. Supporting this hypothesis, the results revealed that greater dCOR was correlated with increased amygdala-centred FC with the left HC and diminished FC with the pgACC in responses to fearful faces (vs. neutral faces). Additionally, exploratory whole-brain analysis found that the cerebellum strengthened FC with the amygdala, whereas the SMG decreased it during processing fearful faces (vs. neutral faces). Especially, the SMG has been reported to have lower FC with the amygdala in anxiety disorder patients^22–25^, although its exact function has yet to be elucidated. The present study is the first to show that unstimulated basal cortisol levels, measured task-irrelevantly in everyday life, is associated with the strength of FC between the amygdala, HC, and pgACC that are implicated in both anxiety and HPA axis regulation^3, 7^. Given that even single basal cortisol levels measurements have been reported to be significantly correlated with total cortisol output^26^, dCOR might reflect a similar component of total cortisol output that is more stable over time than DS or CAR^27^, which would make it a potent predictor for the strength of neural connectivity between HPA-axis regulatory regions, specifically between the amygdala, HC, and pgACC.

Interestingly, the present study further demonstrated a significant mediation effect of amygdala-pHC FC on the positive relationship between dCOR and anxiety symptoms, provided that the effect of amygdala-pgACC FC was factored in as a covariate (although the pgACC itself was not significantly associated with anxiety directly). The pgACC has been implicated in emotional regulation^28^, and its perturbed functional coupling with the amygdala has been substantiated in anxiety disorders^7, 29^. Since the pgACC has been reported to have a regulatory effect on the amygdala responses to emotional stimuli^30, 31^, individuals with lesser connectivity between the amygdala and pgACC during fear-related processing might be susceptible to anxiogenesis as it pertains to greater dCOR. Complicated interactions between the amygdala, HC, and pgACC might contribute to the controversy over the link between cortisol and anxiety disorders^32^.

The R-Amy cluster used as a seed in this study was located on the amygdala’s caudomedial portion^33^, which encompasses its medial nucleus (MeA) and central nucleus (CeA)^34^, both of which are involved in stimulating HPA responses. Of the 2 regions, the CeA, which expresses CRH-producing neurons, is more specifically implicated in generating anxiety-related behaviors and HPA axis activation through its projection to other regions jointly involved in these processes such as the HC and mPFC^35, 36^. CeA-localized glucocorticoids have been reported to exacerbate anxiety-like behaviors^37^ that can be suppressed by MR or GR antagonists in the CeA^38^. Upon encountering a possible threat, greater circulating basal dCOR levels might facilitate a prompt occupation of a relatively limited number of unbound MRs and GRs in the CeA. This remains speculative, however, and further studies are needed to reveal the exact molecular mechanism of the 2 corticosteroid receptors associated with daily cortisol concentrations.

At the level of the CeA and MeA nuclei, the R-Amy was connected with the posterior and anterior parts of the left HC in proportion to dCOR levels, which suggests the importance of contralateral interaction between the R-Amy and the left HC when anticipating a possible threat. Substantial evidence indicates that the R-Amy is more important in emotional face perception^39, 40^. A diffusion-tensor tractography study indicated that a bundle of fibers originating on 1 side of the amygdala can project to the contralateral side, extending to contralateral adjacent regions such as the HC^41^. An anterior part of the left HC is associated with face discrimination and fear-specific emotional processing, and a posterior part of the left HC is generally involved in visual perception processing^42^. In contrast, anterior and posterior portions of the right HC are both linked with a wider range of emotions (not-specific to fear)^42^. Altogether, these findings suggest that the interaction between the R-Amy and left HC might be especially important in fear-related emotional processing and that dCOR might be highly relevant to this interaction.

Besides dCOR, we found a significant effect of CAR on decreased L-Amy FC to the parahippocampal gyrus and right temporal pole (as shown in Supplementary material). No past study has found a significant association of CAR with local activation or FC originated from the amygdala, notwithstanding the assumed relationship between them^43^. In contrast, we did not find a significant effect of DS on amygdala-centred FC, despite the report that normative diurnal decline was associated with diminished amygdala activity and enhanced mPFC activity in older adults (>60 years old)^20^. The diurnal cortisol variation in young adults might be different from that in older adults who are susceptible to its perturbation due to aging^44^. Additionally, a resting-state fMRI study found a significant positive correlation between pre-scan cortisol levels and diminished FC between the amygdala and pgACC^19^, partly conforming with the current findings on the correlation between greater dCOR and diminished amygdala-pgACC FC. Unstimulated baseline cortisol levels might be a steady biomarker for neural connectivity strength between the amygdala and the pgACC as well as HC.

This study’s limitations should be considered when interpreting the results. First, we used cortisol measurements from 3 time points for 2 consecutive days. Particularly, CAR was measured by 2 points sampling, but not 3 points (or more) sampling as recommended^45^. The results of CAR were thus reported as additional information. Second, we did not use an objective method of verifying awakening time (e.g., actigraphy or electrocardiography) or verifying sampling times (e.g., an electronic monitoring system), although a previous study using polysomnography found no differences between self-reported awakening times and objectively recorded awakening times^46^. However, these 2 limitations are unlikely to considerably undermine the results of dCOR, which is relatively unaffected by awakening-related factors as compared to CAR. Third, the sample is relatively small, although it was still reasonably large compared to previous studies focusing on unstimulated cortisol (*n* < 30)^18, 19^. Finally, although the assessment of anxiety symptoms preceded MRI scans and saliva collection in this study, the causality between amygdala-centred connectivity, cortisol, and anxiety remains to be unraveled. Future studies examining larger samples with longitudinal measurement and different age tiers will further delineate the precise chronological relationships between cortisol, anxiety, and amygdala-HC-pgACC connectivity and the complicated mechanism behind the relationship between cortisol and emotional dysregulation.

In conclusion, we provide the first evidence that daily cortisol concentrations, measured task-irrelevantly in everyday life, is associated with the strength of amygdala-centred FC with the HC and pgACC that are implicated in anxiogenesis and HPA axis regulation. Notably, FC strength with the left HC at the seed of the central and medial nuclei of the right amygdala has a strong correlation with daily cortisol concentrations. Furthermore, amygdala-HC FC mediated the relationship between cortisol and anxiety, provided that the effect of amygdala-pgACC FC strength, a presumable neural indicator of individual emotional regulation, was taken into account. Individuals with lesser connectivity between the amygdala and pgACC during fear-related processing might be more vulnerable to anxiogenesis as it pertains to greater circulating cortisol secretion. Individual functional patterns of the amygdala-hippocampal-pgACC neural circuit might provide a key to better understand the complicate link between cortisol and anxiety-related behaviors. Future studies are needed to elucidate the mechanism behind the relationship between cortisol, anxiety, and amygdala-HC-pgACC neural circuit.

## Methods

### Participants

The Kitasato University Hospital Institutional Review Board approved the study (approval number: C11–690). All experimental methods were carried out in accordance with the ethical guidelines determined by the National Ministry of Health, Labour and Welfare and the Declaration of Helsinki. All participants provided written informed consent. Forty-one healthy participants were recruited via advertisements in a local magazine and billboards at Kitasato University [all right handed, 25 women, mean age = 21.9 years, range = 20–33, standard deviation (SD) = 2.9). The eligibility criteria were (1) right-handedness as defined by the Edinburgh Handedness Inventory^47^, (2) no Axis-I psychiatric disorders or substance-abuse history as determined using the Mini-International Neuropsychiatric Interview^48^, (3) no major medical illnesses, (4) no regular intake of psychotropics, steroids, or opioids, (5) no head injury with loss of consciousness, and (6) no history of habitual smoking. No one was engaged in shift work or had jet lag. The participants overlapped with those of our previous study examining a cognitive measure’s neural correlates^33^. In this study, however, we focused on completely different hypotheses, and the analyses reported here do not overlap with those previously published.

### Study procedures

All participants completed MRI scans and saliva collection, which were generally conducted within 2 weeks after the initial assessment. Thirty-two percent of them gathered their saliva prior to MRI scans (average interval between saliva collection and MRI scans ± SD = 3.8 ± 13.8 days). At the initial assessment, data were obtained concerning age, sex, body mass index (BMI), monthly alcohol consumption, and years of education in order to account for their possible confounding effects on cortisol^45^. Women were asked to disclose the presence of menstrual irregularity, their typical menstrual period, and last menstruation date. None used oral contraceptives. The scoring methods for BMI, monthly alcohol consumption, and menstrual period are detailed in the Supplementary material.

### Psychological assessment

Psychological distress including anxiety and depression was assessed at the first assessment using the Hopkins symptom checklist (HSCL)^49^, which is reported to be closely associated with HPA axis function^50^. We used the Japanese version of HSCL, whose reliability and validity have been established^51, 52^. The HSCL is a self-report questionnaire measuring distress experienced during the past week across five dimensions: (1) somatization, (2) obsessive–compulsive behavior, (3) interpersonal sensitivity, (4) anxiety, and (5) depression. These symptoms were rated for 54 items on a 4-point frequency scale (i.e., from 1: “Not-at-all” to 4: “Frequently”). Mean and SD of each subscale is shown in Table S2.

### Saliva collection and cortisol assay

Salivary cortisol was measured 3 times daily: upon awakening (T1), 30 min after awakening (T2), and at bedtime (T3). At the first meeting, an experimenter fully explained the procedures at each time point and demonstrated saliva collection. Participants were encouraged to practice taking their saliva using a sample tube and to read a collection protocol at home. Participants collected saliva on 2 consecutive typical weekdays using personalised kits with 6 tubes containing Salimetric Oral Swabs (Salimetrics, Inc., State College, PA), each labeled in a unique colour with date and time of measurement (e.g., *The 1st day, at bedtime*). Participants were allowed to decide which day they would start saliva collection provided it was a typical weekday within 2 weeks after the initial assessment.

During the consecutive 2 days and 1 night before starting, alcohol was strictly prohibited. Firm restrictions also applied to consuming any food or drink besides water, exercising, tooth-brushing, and showering or bathing within 30 min after awakening or 1 h before bedtime. At awakening, participants were required to record the time they went to bed and awoke and to rate sleep quality and perceived stress on a 4-point scale (i.e., 0–3). Within 24 h after completing saliva collection, the refrigerated samples were transported to the National Institute of Occupational Safety and Health.

For the salivary assay, slowly thawed samples were centrifuged at 3000 rpm (G-force = 1710) for 15 min. Salivary cortisol concentrations were determined using enzyme-linked immunoassay kits (IBL International, Hamburg, Germany). Inter-assay and intra-assay concentration variations were below 7.3% and 9.3%, respectively. Because the heterogeneity of cortisol measures prevented previous findings from converging, we used the 3 major cortisol indices: cortisol awakening response (CAR), diurnal slope (DS), and daily cortisol concentrations (dCOR). As for CAR, however, the findings were reported in Supplementary material because 3 (or more) time points sampling is recommended for the assessment of CAR^45^. Based on the previous formula^53^, these 3 measures were calculated as follows: [(T1 + T2) × 0.5 h / 2] – [T1 × 0.5 h] for CAR (i.e., the area under the curve with respect to increase), (T3 – T1) for DS, and the sum of 3 time points for dCOR. Additionally, the area under the curve with respect to ground (AUCg) was reported as a reference index of dCOR: [(T1 + T2) × Time_T2-T1 (h)_ / 2] + [(T2 + T3) × Time_T3-T2 (h)_ / 2]. Each measure was averaged across the 2 collection days. The cortisol measures were square-root transformed if the Shapiro-Wilk test indicated that they were not normally distributed.

### fMRI experimental design

**Figure 5:**
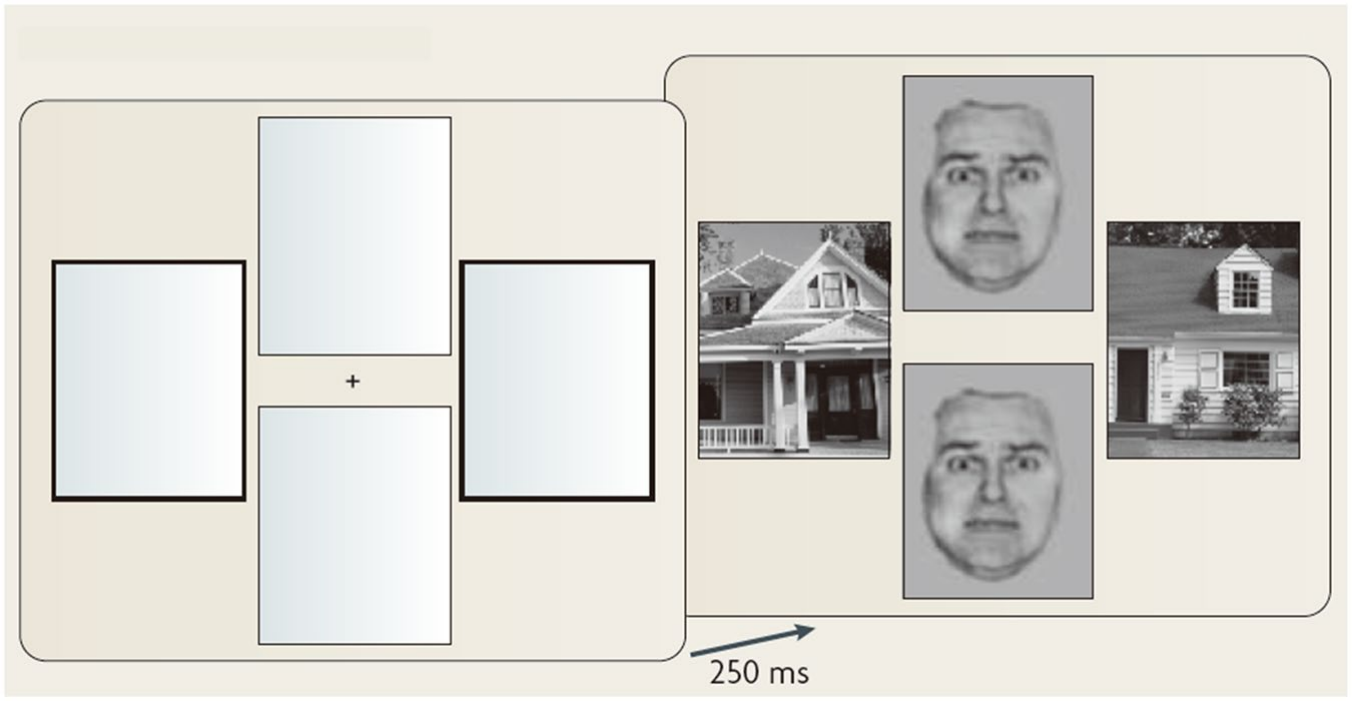
Experimental paradigm. The first display presents 4 empty frames in vertically and horizontally paired positions, with either the 2 horizontal or the 2 vertical frames being highlighted. The next display shows a central fixation point followed by paired faces and paired houses arranged in vertical or horizontal positions. The 2 highlighted frames always predict the locations of the paired houses. Both faces had either neutral or fearful expressions. The positions of the paired faces or houses (vertical or horizontal) and the facial expressions (neutral or fearful) were counterbalanced across subjects and were presented in a random order. The figure is reprinted with permission from the ref. 55 (2010) © Macmillan Publishers Ltd., in Nature Publishing Group. The figure is a modified version of the original figure from the ref. 54 (2001) © Cell Press.

The emotional faces perception task was a modified version of the face-house matching task^54^ (Fig. 5). The task procedures were described elsewhere^33^. Briefly, after a 2000-ms visual display consisting of 4 empty frames in vertically- or horizontally-paired positions with either the 2 horizontal or the 2 vertical frames being highlighted, a fixation point was presented at the screen’s center for 1000 ms. Next, a pair of faces and a pair of houses were presented at the frames’ locations for 250 ms. Paired houses were always preceded by the 2 highlighted frames, and participants were instructed to attend to these highlighted frames and subsequent houses. Fearful faces that emerge outside the focus of attention, as compared to neutral faces, are known to reliably elicit responses of the amygdala^55^. The positions of the paired faces or houses (vertical or horizontal) and the types of facial expressions (neutral or fearful) were presented in a random order for each subject. The stimulus intervals were jittered between 3500 ms and 12500 ms for the 48 trials with a mean of 7500 ms. The task duration was 8 min 51 sec (including dummy scans).

### fMRI acquisition

Anatomical and functional MRI scans were acquired using a 1.5-tesla GE Signa scanner (Signa HDxt; GE Healthcare, Wauwatosa, WI) with an 8-channel phased-array head coil. Structural images were acquired with a 3D T1-weighted sequence (slice thickness without slice gap = 1.2 mm, field of view (FOV) = 240 mm, matrix = 288 × 256, repetition time (TR) = 13.5 ms, echo time (TE) = 5.8 ms, and flip angle (FA) = 20°). For functional images, data were acquired using fast-gradient-echo-planar T2*-weighted imaging with 5 dummy volumes at the beginning of the session. Each functional volume consisted of 30–34 transverse slices (slice thickness = 4.0 mm, slice gap = 1.0 mm, FOV = 260 mm, matrix = 128 × 128, TR = 3,000 ms, TE = 40 ms, and FA = 90°).

### fMRI preprocessing

We used CONN Functional Connectivity Toolbox version 16a^56^; http://www.nitrc.org/projects/conn) that was implemented in SPM12 (Institute of Neurology, University College London, London, UK; http://www.fil.ion.ucl.ac.uk/spm/software/spm12) for preprocessing and FC analyses. Preprocessing procedures consisted of: segmentation, slice-time correction, coregistration, spatial normalization to the Montreal Neurological Institute (MNI) space, Gaussian spatial smoothing (full width at half maximum = 6 mm), and outlier detection (or ‘scrubbing’). In addition, the following confounders were removed by the principal component-based noise-correction ‘CompCor’ method^57^: signal noises from the white matter and cerebrospinal fluid, within-subject covariate including head motion artifacts and scrubbing parameters, and the main experimental condition effects convolved with haemodynamic response function. Band-pass filtering was applied with a frequency window of 0.01 to 0.12 Hz.

### FC analyses

#### Definition of the amygdala as seed region

We set the amygdala as a seed region. The seed was extracted from clusters showing significant responses to fearful faces (vs. neutral faces) [left amygdala (L-Amy) = −21 −10 −17, 351 mm^3^, right amygdala (R-Amy) = 21 −10 −17; 189 mm^3^; both significant at *p* < 0.001 (uncorrected) within the amygdala mask defined by the Wake Forest University (WFU) PickAtlas^58^]^33^ (the left amygdala cluster was also significant at FWE-corrected *p* < 0.05 with small volume correction).

#### Exploratory whole-brain analysis

Using these amygdala seeds, we first performed a whole-brain analysis to explore a possible association between cortisol indices and different brain regions including the HC and vmPFC. These target brain regions were defined according to a default atlas in CONN [132 regions of interest (ROIs)] that comprised cortical and subcortical areas in the FSL Harvard-Oxford atlas (http://www.cma.mgh.harvard.edu/fsl atlas.html) and cerebellar areas in the Automated Anatomical Labeling (AAL) atlas (http://www.gin.cnrs.fr/AAL). The time series of blood-oxygen-level dependent (BOLD) signal values were averaged across all voxels within each target region. Bivariate-regression analyses were conducted to determine the linear association of the BOLD time series between the amygdala seed and target regions, creating FC maps for each participant. Each cortisol index was entered into a general linear model as an independent variable explaining the FC as a dependent variable. Statistical significance was defined as *p* < 0.05 (2-sided), with false-discovery rate (FDR) corrections for multiple tests.

#### Seed-to-voxel analysis

When the brain regions were observed to be significantly connected with the amygdala in the whole-brain analysis, we conducted a seed-to-voxel analysis to clarify the connected regions’ locations. Due to the above-mentioned hypothesis, we adopted clusters found in the HC and vmPFC within anatomical masks of the HC, mOFC, and ACC in the WFU PickAtlas. Height threshold was set at *p* < 0.001 with extent threshold *k* > 15 voxels.

### Regression analysis: amygdala-centred FC and anxiety/depression

Further, regression analyses were performed to examine the effect of anxious and depressive mental states (as measured by HSCL) on the amygdala-centred FC. The estimated values (βs) of amygdala-centred FC with the HC, vmPFC or the other brain regions that were shown to be significant in the whole-brain analysis (whose clusters were specified by the seed-to-voxel analysis) were extracted with MarsBar 0.43 (http://marsbar.sourceforge.net/). These amygdala-centred FC values were fed into a step-wise regression analysis with each of the 5 HSCL subscales as a dependent variable using SPSS version 24.0 J (IBM, Inc., Tokyo, Japan). Statistical significance was defined as *p* < 0.05 (2-tailed).

### Mediation analysis: an effect of amygdala-centred FC on the relationship between cortisol and anxiety/depression

Provided that amygdala-centred FC with the HC and vmPFC were significantly correlated with both cortisol and anxiety/depression, we further investigated whether the amygdala-centred FC would mediate the relationship between cortisol and anxiety/depression using PROCESS implemented in SPSS (http://processmacro.org/index.html). We estimated the bootstrapped (5,000 samples) 95% confidence intervals for the indirect effect. Statistical significance was defined as *p* < 0.05. Since psychological assessments preceded MRI acquisition and saliva collection, the independent variable was anxiety/depression (independent variable = X), and the dependent variable was cortisol (dependent variable = Y). Amygdala-centred FCs were assigned as the mediator (M). Additionally, when both an amygdala-HC FC and amygdala-vmPFC FC were associated with cortisol, but only 1 was correlated with anxiety/depression, the anxiety/depression-correlated FC was entered in the mediation model as M. The other (anxiety/depression-uncorrelated) FC could be incorporated as a covariate to improve the variance percentage explained for Y, because amygdala-HC FC and amygdala-vmPFC FC are presumed to function antagonistically to each other^30, 31^. A mediation effects’ presence was considered confirmed if (1) we would observe a statistically significant indirect effect of the variable X on Y through the mediator M, and (2) the direct effect would be reduced or nullified (i.e., partial or complete mediation, respectively) in the mediation model (as compared to the direct effect in the initial model excluding M).

### Data availability statement

The data that support the findings of this study are available on request from the corresponding author [Y.H.], as long as the data sharing complies with the Japanese Ministry of Health, Labor and Welfare Ethical Guidelines. The data are not publicly available due to them containing information that could compromise research participant consent.

## Electronic supplementary material

SupplementaryInfo
