# Supplementary material for: Amygdala-centred functional connectivity affects daily cortisol concentrations: a putative link with anxiety
Source: Sci Rep. 2017 Aug 16;7:8313. doi: 10.1038/s41598-017-08918-7 (PMC5559590; doi:10.1038/s41598-017-08918-7)
Supplement: Supplementary file 1 — SupplementaryInfo [file 41598_2017_8918_MOESM1_ESM.pdf]

Amygdala-centred functional connectivity affects daily cortisol concentrations: a putative link with anxiety

Yuko Hakamata, Shotaro Komi, Yoshiya Moriguchi, Shuhei Izawa, Yuki Motomura, Eisuke Sato, Shinya Mizukami, Yoshiharu Kim, Takashi Hanakawa, Yusuke Inoue, and Hirokuni Tagaya.

Supplementary information

1. Materials and methods

1.1. Demographics

***Body mass index (BMI)*:** BMI was calculated based on this formula: weight (kg) / ([height (m)]^2).

***Monthly alcohol consumption*:** The unit of alcohol for each participant was calculated using the UK National Health Service formula (http://www.nhs.uk/Livewell/alcohol/Pages/alcohol-units.aspx#_blank): volume consumed (ml) * alcohol by volume (%) / 1000. Drinking frequency per month (days) was weighted on the calculated unit of alcohol. For example, for a person who drinks a standard glass (175 ml) of wine (12 %) once a week, the monthly alcohol unit can be calculated as follows: (175 * 12 / 1000) * 4 (days per month) = 8.4, while the alcohol unit is 16.8 for a person who drinks a standard glass of wine twice a week.

***Menstrual period*:** Menstrual status at the start of saliva collection was estimated according to the Ogino method, which has been used for infertility treatment and contraception (Ogino, 1934; Tietze and Lincoln, 1987). In this method, the “ovulatory phase” is presumed to occur between 2 days before and 3 days after the 14th day before the next anticipated menstruation start date. The periods before and after the ovulatory phase were regarded as the “follicular phase” and “luteal phase,” respectively. Women who menstruated irregularly (e.g., menstruation occuring

fortnightly for a month, while later occurring several months after the previous one) were placed in the “irregular cycle” category.

2. Results

2.1. Saliva cortisol and psychological data

No cortisol measure was significantly associated with possibly confounding quantitative variables including age, BMI, monthly alcohol consumption, years of education, sleep duration, sleep quality, or perceived stress at awakening (**Table S1**). For the time of awakening, participants were classified by whether they woke before or after 7:00 AM, as in previous studies (Edwards et al., 2001; Kudielka and Kirschbaum, 2003). Independent samples t-testing showed no significant differences in any cortisol measure between the 23 early awakeners and 18 late awakeners (dCOR: $t(39) = –0.22$, $p = 0.83$; dCOR AUCg: $t(39) = 0.44$, $p = 0.66$; CAR: $t(39) = 0.45$, $p = 0.65$; DS: $t(39) = –0.12$, $p = 0.91$). For the sex effect, independent samples t-testing showed no significant differences in any cortisol measure between the 16 men and 25 women (dCOR: $t(39) = 0.02$, $p = 0.98$; dCOR AUCg: $t(39) = 0.16$, $p = 0.88$; CAR: $t(39) = 0.78$, $p = 0.44$; DS: $t(39) = –0.39$, $p = 0.70$). As for menstrual status, 25 women were classified into 5 groups: “during menstruation” (N = 3), “follicular phase” (N = 8), “ovulatory phase” (N = 5), “luteal phase” (N = 4), and “irregular cycle” (N = 5). No significant differences between these groups were found in analysis of variance (ANOVA) (dCOR: $F(4,20) = 0.91$, $p = 0.48$; dCOR AUCg: $F(4,20) = 0.82$, $p = 0.53$; CAR: $F(4,20) = 0.74$, $p = 0.58$; DS: $F(4,20) = 0.90$, $p = 0.48$).

2.2. Whole-brain analysis: cortisol awakening response (CAR) and amygdala-centered functional connectivity

Whole-brain analysis showed that larger CAR size was significantly associated with decreased L-Amy-centered FC with an anterior part of the left parahippocampal gyrus ($T = –4.40$; and FDR whole-brain corrected $p = 0.009$) and the right temporal pole ($T = –4.23$, FDR-corrected $p = 0.009$) upon presentation of fearful faces (vs. neutral faces).

Table S1. Correlation coefficients between cortisol measures and potential confounders

	dCOR	AUCg	CAR	DS
dCOR	—			
AUCg	0.96***	—		
CAR	0.10	0.30	—	
DS	0.78***	0.64***	-0.27	—
Age	0.12	0.08	0.08	0.23
BMI	-0.12	-0.14	-0.19	0.03
Years of education	0.22	0.24	0.12	0.25
Monthly alcohol units	0.05	0.02	0.12	0.09
Sleep hours	-0.03	-0.13	0.18	-0.16
Sleep quality	-0.12	-0.07	0.22	-0.19
Perceived stress	0.05	0.04	0.05	0.15

***$p < 0.001$.

Abbreviations: dCOR, daily cortisol concentrations; AUCg, area under the curve with respect to ground (3 time points); CAR, cortisol awakening response (i.e., area under the curve with respect to increase); DS, diurnal slope; BMI, body mass index.

Table S2. Mean and SD of HSCL subscales

	All participants (N = 41)				Men (N = 16)				Women (N = 25)			
	Mean	SD	Min	Max	Mean	SD	Min	Max	Mean	SD	Min	Max
somatization	19.88	5.75	14	43	19.13	3.58	14	28	20.36	6.81	14	43
obsession and compulsion	18.41	7.01	9	35	21.00	7.88	9	35	16.76	5.98	10	31
interpersonal sensitivity	17.49	6.65	10	39	18.13	6.62	11	34	17.08	6.78	10	39
anxiety	12.54	5.58	8	32	12.69	4.84	8	23	12.44	6.10	8	32
depression	20.78	7.33	13	43	20.63	6.30	13	31	20.88	8.04	13	43

Abbreviations: HSCL, Hopkins Symptom Checklist; SD, standard deviation; Min, minimum value; Max, maximum value.

References

Edwards, S., Evans, P., Hucklebridge, F. and Clow, A. 2001. Association between time of awakening and diurnal cortisol secretory activity. Psychoneuroendocrinology 26, 613-622.

Kudielka, B. M. and Kirschbaum, C. 2003 Awakening cortisol responses are influenced by health status and awakening time but not by menstrual cycle phase. Psychoneuroendocrinology 28, 35-47.

Ogino, K. 1934. Conception Period of Women. Medical Arts Publishing Company, PA, USA.

Tietze, S. L., and Lincoln, R. (Eds). 1987. Fertility Regulation and the Public Health. Springer-Verlag, NY, USA.
